# Antibody structure and engineering considerations for the design and function of Antibody Drug Conjugates (ADCs)

**DOI:** 10.1080/2162402X.2017.1395127

**Published:** 2017-11-20

**Authors:** Ricarda M. Hoffmann, Ben G. T. Coumbe, Debra H. Josephs, Silvia Mele, Kristina M. Ilieva, Anthony Cheung, Andrew N. Tutt, James F. Spicer, David E. Thurston, Silvia Crescioli, Sophia N. Karagiannis

**Affiliations:** a St. John's Institute of Dermatology, School of Basic & Medical Biosciences, King's College London, Tower Wing, Guy's Hospital, London, United Kingdom; b NIHR Biomedical Research Centre at Guy's and St. Thomas's Hospitals and King's College London, King's College London, London, United Kingdom; c School of Clinical Medicine, University College London Medical School, London, United Kingdom; d School of Cancer & Pharmaceutical Sciences, King's College London, Guy's Hospital, London, United Kingdom; e Breast Cancer Now Research Unit, School of Cancer & Pharmaceutical Sciences, King's College London, Guy's Cancer Centre, London, United Kingdom; f Institute of Pharmaceutical Science, King's College London, Britannia House, London, United Kingdom; g Femtogenix Ltd, BioPark, Welwyn Garden City, Hertfordshire, United Kingdom

**Keywords:** Antibodies, Antibody Drug Conjugate (ADC), biodistribution, effector functions, Immunoglobulin Fc, IgG1, IgG4, kinetics, stability

## Abstract

Antibody-drug conjugates (ADCs) are emerging as effective tools in cancer therapy, combining the antibody's exquisite specificity for the target antigen-expressing cancer cell together with the cytotoxic potency of the payload. Much success stems from the rational design of “toxic warheads”, chemically linked to antibodies, and from fine-tuning the intricate properties of chemical linkers. Here, we focus on the antibody moiety of ADCs, dissecting the impact of Fab, linkers, isotype and Fc structure on the anti-tumoral and immune-activating functions of ADCs. Novel design approaches informed by antibody structural attributes present opportunities that may contribute to the success of next generation ADCs.

## Introduction

**Antibody-Drug Conjugates and known mechanisms of action in oncology**

The treatment of cancer remains a formidable challenge.^1^ For many tumors, chemotherapy achieves significant clinical benefit, however these agents suffer from lack of specificity for cancer cells and high toxicity, often resulting in adverse effects, poor quality of life, early discontinuation and reduced clinical efficacy.^1^ Targeted treatments in the form of tumor antigen-specific and checkpoint inhibitor antibodies, envisioned for over 100 years since Ehrlich proposed the concept of “the magic bullet”, have now been established in clinical oncology and have earned their place alongside chemotherapeutic agents and small molecule inhibitors in the care of cancer patients.^1^ However, antibodies targeting tumor-associated antigens also suffer from limitations. These include limited tissue penetrance and blocking target-associated pathways due to intrinsic and acquired resistance.^2^

**Figure 1.**
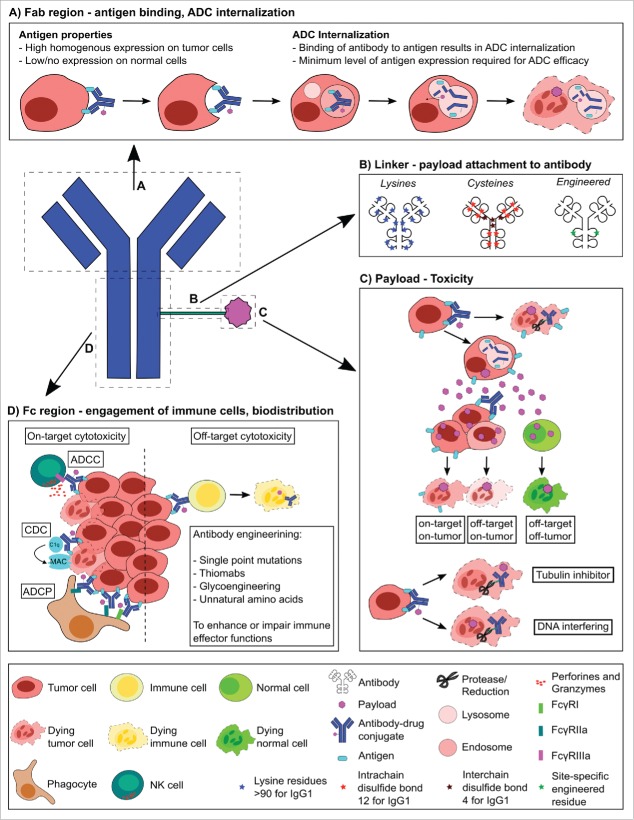
Schematic of ADC components and their role in ADC design, engineering and functions. The Fab region (A) is responsible for antigen recognition and binding, and can lead to ADC internalization. Therefore, the Fab region needs to be targeted to tumor-associated antigens that are homogenously expressed on tumor cells, ideally with little or no expression on normal cells. The payload is attached to the antibody via a cleavable or non-cleavable linker (B). Non-cleavable linkers rely on the complete degradation of the antibody after internalization of the ADC, whereas most cleavable linkers are cleaved by different mechanisms depending on the linker (*i.e.* proteases, reduction) and some cleavable linkers do not depend on ADC internalization for payload release and can result in higher off-target cytotoxicities. The hydrophobicity of linkers can play a vital role in the biodistribution of an ADC. Linkers can be attached non-selectively via lysines or the hinge thiols of cysteines, or antibody engineering can be performed for site-specific linking. The payload (C) is responsible for ADC toxicity and is usually a small hydrophobic molecule, able to cross cell membranes and cause cell death by targeting the cytoskeleton or DNA. Once cleaved from the antibody payloads can enter other (tumor) cells, resulting in further tumor killing (*i.e.* bystander effect) as well as off-target cytotoxicity when entering normal cells. The Fc region of the antibody (D) can trigger immune effector functions such as Antibody-Dependent Cytotoxicity through binding to Fcγ-receptors. However, if the ADC is internalized into non-malignant cells, it can cause off-target cytotoxicity. Antibody engineering can enhance or impair immune effector functions through, for example, single point mutations, Thiomabs, glycoengineering or incorporation of unnatural amino acids.

Antibody-drug conjugates (ADCs) are designed to combine the selectivity of monoclonal antibodies (mAbs) with the cytotoxic potential of chemotherapeutic drugs.^3^ ADCs are tripartite drugs, comprising of a tumor antigen-specific mAb conjugated to a potent cytotoxin via a stable chemical linker.^3^ The three components together give rise to a powerful oncolytic agent, capable of delivering normally-intolerable cytotoxic drugs directly and specifically to cancer cells, guided by the exquisite specificity and high affinity of antibodies for their targets in tumors (Fig. 1).^3^

ADCs have a well-described mechanism of action, namely binding of the mAb to the target antigen resulting in complex internalization through receptor-mediated endocytosis.^4^ Upon fusion of two internalized vesicles, an early endosome is formed whereby cargo is sent through two pathways: recycling which results in trafficking back to the plasma membrane,^5^ or endolysosomal degradation.^4^

The mechanism and location of toxin release depends on the type of linker. Non-cleavable linkers depend on degradation of the antibody with or without a portion of the linker to liberate the toxin from the ADC.^6^ However, cleavable linkers can release toxins through acidic conditions in the lysosome, reduction of the linker in the cytoplasm or cleavage by specific proteases.^6^ For ADCs containing cleavable linkers, the antibody-part of the ADC is either degraded once the toxin is cleaved or is recycled and released outside the cell in vesicles.^4^ Once the toxin is cleaved from the ADC, it enters the cytoplasm and can either bind to its molecular target in the cytoplasm (usually tubulin) or can cross into the nucleus and cause cell cycle arrest and apoptosis by interfering with DNA.^7^ Almost all payloads in clinical development are small hydrophobic molecules, that are able to cross biomembranes once cleaved from the ADC.^8^ Therefore, nuclear DNA as well as the cytoskeleton in the cytoplasm are suitable locations for the payload to interfere with critical cellular mechanisms resulting in cell death.

The majority of ADCs have clinical efficacy through on-target on-tumor effects; however some ADCs also cause off-target on-tumor cell killing, referred to as the bystander-effect.^9^ This effect exploits the ability of payloads to cross cell membranes and exert their cytotoxic effect on neighbouring cancer cells that lack target antigen expression. The payload may be cleaved from the antibody in the tumor microenvironment due to local acidic pH conditions and the presence of proteases released locally by tumor and dead cells.^10^ The bystander effect is especially advantageous for heterogeneous tumors consisting of tumor cells with low or absent target antigen expression that cannot be recognized through the antibody variable regions of the ADC.^7,11^ The majority of ADCs internalize, however there are also non-internalizing ADCs that target tumor cells in the same way but can exert off-target on-tumor effects, making use of the bystander mechanism as one of their primary modes of action. However, this type of ADCs come with a perceived risk of also killing immune cells, such as antigen-presenting cells, that may be important in tumor growth restriction, in an off-target fashion. Since non-internalizing ADCs have had restricted success in the clinic, internalizing ADCs are still at the forefront of the development of targeted anti-cancer therapeutics.^3^

## ADCs in clinical use

After decades of failure with the first approved ADCs being withdrawn from the market due to fatal cytotoxicity,^12^ ADCs are now one of the fastest growing fields of oncology therapeutics. There are currently four FDA- and EMA- approved ADCs within clinical practice with durable clinical responses reported: gentuzumab ozogamicin (Mylotarg™) targeting CD33 expressed by acute myeloid leukemia (AML) has just been re-approved in the US, after being withdrawn in 2010,^13,14^ brentuximab vedotin (Adcetris™) which targets CD30 expressed by Hodgkin lymphoma (HL) and systemic anaplastic large cell lymphoma (ALCL),^15^ trastuzumab emtansine (Kadcyla™, also known as T-DM1) targeting HER2 expressed by 20–25% of breast carcinomas and other solid tumor types^16^ and the recently-approved inotuzumab ozogamicin (Besponsa™) targeting the B-cell lineage marker CD22, expressed on acute lymphoblastic leukemia cells.^17^ (Table 1). Since their approval, there has been significant growth in this class of oncology therapeutics, with over 65 agents in early and late stage clinical trials (see Table 2 for ADCs in Phase I/II, II and III clinical trials).

**Table 1.** Approved ADCs in clinical use.

ADC	Developer	Indication	Target Antigen	Antibody Type/mutations	Linker	Cytotoxin	Year of approval
Gemtuzumab ozogamicin (Mylotarg™)	Pfizer	Acute myeloid leukemia	CD33	Humanised IgG4 IgG4 κ antibody hP67.6	Acid-labile hydrozone bifunctional linker	Calicheamicin	FDA Approved in 2000 in the US, withdrawn in 2010, re-approved in 2017
Brentuximab vedotin (Acetris™)	Seattle Genetics	Relapsed Hodgkin Lymphoma and systemic anaplastic large cell lymphoma	CD30	Chimeric IgG, fusion of the variable heavy and light region of the murine anti-CD30 antibody AC10 with the *constant gamma1-heavy* and *kappa-light* region of the human immunoglobulin	Cathepsin cleavable valine-citrulline approx. DAR 4	MMAE	Accelerated approval by the FDA in 2011, full approval 2015
Trastuzumab emtansine (Kadcyla™)	Roche	HER2+ breast cancer	HER2	Humanised IgG1 (from mouse)	Non-cleavable thioether linker, DAR 3–4	DM1	FDA approved in 2013
Inotuzumab ozogamicin (Besponsa™))	Pfizer	Acute lymphoblastic leukaemia	CD22	Recombinant humanised IgG4	Acid-labile hydrozone	Calicheamicin, CM1	FDA approved in 2017

**Table 2.** ADCs in clinical development in Phase I/II, II or III (U.S. National Institutes of Health. Clinical trials. at https://clinicaltrials.gov).

ADC	Developer	Indication	Target Antigen	Antibody Type/mutations	Linker	Cytotoxin	Phase/Trial Number/Status
Mirvetuximab soravtansine (IMGN853)	ImmunoGen	FRα-positive, epithelial ovarian cancer	Folate Receptor-alpha (FRα	IgG1, humanized	Sulfo-SPDB, DAR 3–4	DM4	III, NCT02631876
Sacituzumab Govitecan (IMMU-132)	Immunomedics (licensed to Seattle Genetics)	Refractory/Relapsed Triple-Negative Breast Cancer	TROP2	IgG1, murine anti-Trop-2 mAb, designated RS7-3G11 (or RS7), was humanized to reduce immunogenicity for clinical use	CL2A, DAR 7.6	SN38	III, NCT02574455
Rovalpituzumab tesirine (Rova-T, SC16LD6.5)	AbbVie (Stemcentrx)	Small cell lung cancers, Relapsed or Refractory Delta-Like Protein 3-Expressing Small Cell Lung Cancer	DLL3	humanised DLL3-specific IgG1 monoclonal antibody SC16	PEG8-val-ala	D6.5 PBD	III, NCT03061812
Depatuximab mafodotin (ABT-414)	AbbVie	Glioblastoma with EGFR Amplification, Glioblastoma Multiforme	EGFRvIII	Humanised recombinant IgG1/κ	Mc	MMAF	IIb/III, NCT02573324
							II, NCT02343406
Vadastuximab talirine (SGN-CD33A)	Seattle Genetics	AML	CD33	Humanised anti-CD33 IgG1, engineered to contain a cysteine at position 239 on both heavy chains, S239C	Mc-val-ala-dipeptide, DAR2	PBD	III, discontinued June 2017 due to higher rate of deaths in patients treated with Vadastuximab talirine compared to control
AGS-16C3F	Agensys/Astelas	Renal cell carcinoma	ENPP3	Human IgG2κ	Mc	MMAF	II, NCT01672775
Anetumab ravtansine (BAY 94–9343)	Bayer HealthCare	Mesothelin expressing tumours	Mesonthelin	Fully human IgG1	Disulfide SPDB	DM4	II, NCT01439152
Coltuximab Ravtansine (SAR3419)	ImmunoGen	Diffuse large B-cell lymphoma	CD19	Humanised IgG1	Disulfide SPDB, DAR 3–5	DM4	II, NCT01472887
Denintuzumab mafodotin (SGN-CD19A)	Seattle Genetics	Diffuse Large B-Cell Lymphoma or Follicular Lymphoma	CD19	Humanized IgG1	Mc	MMAF	II, NCT01786096
DS-8201a	Daiichi Sankyo	HER2+ breast cancer	HER2	Humanised IgG1 (from mouse)	Peg4-maleimide, DAR8	Exatecan	II, NCT03248492
Glembatumumab vedotin (CDX-011)	Celldex Therapeutics	Triple negative breast cancer, Advanced or Metastatic Squamous Cell Carcinoma of the Lung, Recurrent or Refractory Osteosarcoma, Melanoma	Glycoprotein NMB	Human IgG2	Vc-PAB	MMAE	II, NCT01997333
							II, NCT02713828
							II, NCT02487979
							II, NCT02363283, NCT02302339
Indusatumab vedotin (MLN-0264 or TAK-264)	Takeda-Millenium	Gastrointestinal tumour; solid tumours	Guanylyl cyclase C	Human IgG1	Vc-PAB	MMAE	II, NCT02202785
Labetuzumab govitecan (IMMU-130)	Immunomedics	Metastatic Colorectal cancer	CEACAM5	Humanised IgG1 Kappa	CL2A, acid-labile, DAR8	Irinotecan metabolite (SN-38)	II, NCT01915472
Lifastuzumab vedotin (RG-7599 or DNIB0600A)	Genentech/Roche	Ovarian cancer, Non-small cell lung cancer	NaPi2b	Humanised IgG1	Vc-PAB	MMAE	II, NCT01991210 (completed)
Lorvotuzmab mertansine (IMGN901)	ImmunoGen	Leukemia, solid tumours, Small-cell lung cancer	CD56	Humanised IgG1	Disulfide-SPP	DM1	II, NCT02420873
							I/II, NCT01237678, completed
Naratuximab emtansine (IMGN529)	ImmunoGen/Debiopharm	NHL	CD37	Humanized IgG1	SMCC	DM1	II, NCT02592876, NCT02855359
Pinatuzumab vedotin (RG-7593)	Genentech	Follicular B-cell non-Hodgkin's lymphoma	CD22	Humanised IgG1	Vc-PAB	MMAE	II, NCT01691898
Polatuzumab vedotin (RG-7596, DCDS4501A)	Genentech/Roche	Non-Hodgkin's lymphoma DCBCL and follicular	CD79b	Humanised IgG1	Vc-PAB	MMAE	II, NCT01691898,
							Ib/II, NCT02729896
PSMA ADC	Progenics/Seattle Genetics	Prostate Cancer	PSMA	Fully human IgG1	Vc	MMAE	II, NCT01695044
SAR566658	Sanofi	TNBC	CA6	Humanized IgG1	SPDB	DM4	II, NCT02984683
BMS-986148	Bristol-Myers Squibb	Advanced solid tumours	Mesothelin	Human IgG1 wild type antibody	Undisclosed	Undisclosed	I/IIa, NCT02341625
CDX-014	Celldex	Renal cell carcinoma	TIM1	Fully human mAb IgG1κ (clone 2.70.2)	Vc	MMAE	I/II, NCT02837991
Humax-Axl-ADC	Genmab	Multiple solid tumours	AXL	IgG1	Vc	MMAE	I/II, NCT02988817
Indatuximab Ravtansine (BT-062)	BioTest	Multiple myeloma	CD138	Chimeric IgG4	Disulfide SPDB	DM4	I/IIa, NCT01638936
Milatuzumab doxorubicin (IMMU-110 or hLL1-DOX)	Immuno-medics	Chronic lymphocytic leukaemia; multiple myeloma; non-Hodgkin's lymphoma	CD74	Humanised IgG1	Hydrazone	Doxorubicin	I/II, NCT01585688
SAR408701	Sanofi	Solid tumours	CEACAM5	IgG1	SPDB	DM4	I/II, NCT02187848
Tisotumab vedotin (HuMax-TF-ADC)	Genmab	Multiple solid tumours	Tissue factor (CD142)	Human IgG1	Vc-PAB	MMAE	I/II, NCT02001623, NCT02552121
U3-1402	Daiichi Sankyo	Human Epidermal Growth Factor Receptor 3 Positive Metastatic Breast Caner	HER3+	Humanized anti-HER3 IgG1	Peptide linker, DAR 7–8	DX-8951 derivative, topoisomer-ase I inhibitor	I/II, NCT02980341

AML – acute myloid leukemia; DLL3 – delta like protein 3; DM1/DM4 – maytansonoid derivatives; Mc – maleimidocapryl; MMAE/MMAF – monomethyl auristatin E/ monomethyl auristatin F; PBD – pyrrolobenzodiazepine; PEG8 – polyethylenglycol 8; PSMA – prostate specific membrane antigen; SMCC – succinimidyl-4-(*N*-maleimidomethyl)cyclohexane-1-carboxylate; SN38 – irinotecan prodrug; SPDB – *N*-hydroxysuccinimidyl 4-(2-pyridydithio)butanoate; Vc – valine-citrulline; SPP *N*-succinimidyl 4-(2-pyridyldithio)pentanoate sulfo-SPDB – *N*-hydroxysuccinimidyl 4-(2-pyridyldithio)-2-sulfobutanoate; val-ala – valine- alanine

Two of the approved ADCs, brentuximab vedotin and trastuzumab emtansine, use auristatin- and maytansinoid-based warheads, respectively. These microtubule-targeting agents represent two-thirds of all clinical stage ADC payloads.^18^ Mirvetuximab soravtansine (IMGN853, Phase III) and Depatuximab mafodotin (ABT-414, Phase IIb/III) also fall into this category line, consisting of the maytansinoid warhead DM4, similar to DM1, the drug component of trastuzumab emtansine^19^ and MMAF, respectively. The other three ADCs in Phase III clinical trials (Sacituzumab Govitecan, Rovaltuzumab tesirine, Vadastuximab talirine) consist of drugs that target DNA and are therefore cytotoxic to both proliferating and non-proliferating cells (Table 2). SGN-CD33A (recently discontinued)^20^ and rovalpituzumab tesirine (Rova-T™) consist of pyrrolobenzodiazepines (PBDs),^21,22^ payloads designed to interfere with the minor groove of the DNA. The drug component of IMMU-132 is SN38a camptothean analogue, which inhibits DNA Topoisomerase I.^23,24^ Generally, there is a trend towards payloads with higher potency featuring IC_50_s in the nanomolar and sub-nanomolar scale.^1^ Despite many advances, finding novel payloads with optimal *in vivo* tolerability and high therapeutic index is still an ongoing challenge. New classes of toxins, evaluated in pre-clinical settings, such as the DNA alkylating agent indolinobenzodiazepine pseudodimers (IGNs), resulted in promising ADCs characterized by both high *in vivo* tolerability and high therapeutic indices and has been now moved forward to clinical evaluation.^25^

Over the past decade, much research has been conducted focusing on linker chemistry and optimizing cytotoxic payloads. However, while the antibody is often viewed as a mechanism to deliver the cytotoxic payload, other potential effects pertaining to the antibody's unique structural characteristics, remain less-well elucidated. In this review, we consider the ADC as a tripartite drug, specifically focusing on the underexplored antibody rather than the toxic payload portion or the linker.

## The Fab regions: Selection of, and specificity to, target expression of antigen

The successful development of an ADC is dependent on the premise of selecting an appropriate target antigen, with its suitability forming a key determinant of the efficacy of the ADC. Targeted therapies such as ADCs exploit the difference in protein expression between cancer cells and normal cells in order to select a suitable target antigen. Current targets being exploited include HER2 (*i.e.* trastuzumab emtansine)^26^ and CD30 (*i.e.* brentuximab vedotin).^27^ It is widely accepted that the target antigen should be homogeneously and selectively expressed on the surface of tumor cells with little or no expression on normal tissues in order to limit on-target off-tumor toxicity.^28^

Target validation for ADCs must be based upon the reliable identification of target antigens. Genomic studies have highlighted the complex heterogeneity in tumors with different cell sub-populations harboring distinct phenotypic diversity resulting from the integration of genetic and non-genetic influences that define intra-tumor and inter-tumor heterogeneity.^29^ This represents a profound challenge for antigen selection and, combined with sampling bias, may negatively impact drug discovery and validation of suitable antigen targets for ADCs.^29^ While homogeneous target antigen expression is not an absolute requirement for ADC efficacy, since heterogeneous tumors may benefit from bystander killing, the most advanced ADCs within clinical development are for hematological indications which have a largely consistent expression of lineage specific markers (*i.e.* CD22, CD30)^30^, such as CD79b (RG-7596, DCDS4501A). These lineage specific markers of hematologic cells are also targets for three out of four approved ADCs: CD22 (Besponsa™) for the treatment of acute lymphoblastic leukemia, CD30 (Acetris™) for the treatment of relapsed Hodgkin Lymphoma and systemic anaplastic large cell lymphoma and CD33 (Mylotarg™) for the treatment of acute myeloid leukemia (Table 2).^31^

Traditionally, interest in target antigens has largely focused on those expressed on tumor cells. However, there has been growing interest in targeting antigens present within the tumor microenvironment, including those within the neovasculature, sub-endothelial extracellular matrix and the tumor stroma.^32–34^ ADCs that target the stroma cause tumor cell death by reducing the concentration of growth factors produced by the stroma.^35^ Since all tumor cells are dependent on angiogenesis and stromal factors for their survival, ADCs that target such tissues may have a broader efficacy. This is particularly attractive due to the fact that, unlike cancer cells, these cells are genomically stable, and are less likely to develop mutation-related drug resistance.^3^

The target antigen should be well internalized by receptor-mediated endocytosis and should not be down-regulated by endocytosis or by the effects of repeated stimulation during treatment.^1,36^ In order for an ADC to generate a clinical effect, antibody recognition of its epitope on the antigen must result in endocytosis.^37^ In general, antigens that internalize well, with low expression on normal tissue and high expression on tumors are preferred for an ADC approach as they minimize potential toxicity through unwanted on-target, off-tumor expression. However the results of clinical trials indicate it may be difficult to predict the toxicity based on target expression in healthy tissue and toxicities due to off-tumor on-target expression can occur.^38^ In the case of glembatumumab vedotin that targets the transmembrane glycoprotein NMB (gpNMB) on cancer cells, development of skin rash was one of the observed dose-limiting toxicities, which is likely due to membrane expression of gpNMB in epithelial cells of the skin. Previously the development of an ADC directed against CD44v6 was discontinued due to severe skin toxicity linked to high CD44v6 expression in the skin.^39,40^ Interestingly, unlike findings with unconjugated antibody functions, current experimental evidence generally suggests that the antigen density does not directly correlate with the efficacy of the ADC.^41^ Studies of lymphoma, melanoma and prostate cancer have demonstrated no direct correlation between antigen density and therapeutic response for ADCs, and findings suggest that a minimum antigen expression threshold is required for ADC efficacy.^31,42,43^

## The location and roles of the linker: Considerations for conjugation strategies

The linker plays a central role in connecting the cytotoxic agent to the antibody structure. One of the key functions of the linker is to maintain complex stability in the blood circulation, while allowing toxin release upon ADC internalization by target cells.

The first generation of ADCs mostly relied on linking via the antibody's lysine or cysteine residues.^6^ IgG1 isotype antibodies consist of approximately 90 lysine residues, however, due to structural constrains, only 30 can be modified.^8^ Therefore, linking the antibody to the toxin via the lysine residues results in heterogeneous ADCs with regards to both the number of toxins conjugated per antibody (drug-to-antibody ratio, DAR) and the positions of conjugation within the structure. Furthermore, monitoring changes on the antibody scaffold may be challenging, since, in addition to batch-to-batch variations, ADCs within one batch could differ significantly.^44^ Therefore, novel methods are being developed to improve protein characterization and control protein modification processes.^45^ Nevertheless, Kadcycla™ would not be a product unless regulatory authorities were satisfied that the conjugate product could be made consistently.

When conjugating to cysteine residues, disulfide bonds within the antibody must be reduced in order to produce free thiols for conjugation.^46^ IgG1 antibodies consist of 4 inter-chain disulfide bonds – two connecting heavy and light chains and two connecting the two heavy chains in the hinge region, keeping the two half-antibodies together.^47^ There are also 12 intra-chain disulfide bonds, however it has been shown that mild reduction of the antibody with DTT (Dithiothreitol) or TCEP (Tris(2-Carboxyethyl)phosphine) will result in inter-chain bonds being reduced without having an impact on the intra-chain disulfide bonds of the antibody.^48^ However, one of the pitfalls of partial antibody reduction may be loss of the light chain, which can impair the binding properties of the ADC as well as its antigen cross-linking properties which may be key to facilitating internalization.

The location of the linker on the antibody structure could also negatively impact ADC functions. If the linker is conjugated near the Fab antigen binding site, this may interfere with or completely block antigen recognition and hence targeting of the ADC to tumors. Conjugation at or near the Fc regions, may also hinder binding to the neonatal Fc receptor (FcRn) and/or Fc receptors on immune effector cells, or alter antibody folding and structure. Either individually or collectively, these could substantially modify pharmacokinetics, pharmacodynamic properties, target recognition and engagement, effector functions and consequently bioavalability and efficacy. Quantifying the impact of these effects may be particularly complex for heterogeneous ADC preparations designed from lysine or cysteine bound linkers.

Despite drawbacks, cysteine and lysine conjugated heterogeneous ADC products with complex structural characteristics and variable DARs have facilitated ADC design and clinical translation. Current trends are focused on: designing homogenous ADC products with defined DARs via site-specific conjugation;^8^ increasing the polarity and decreasing hydrophobicity of linkers which can provide improved pharmacokinetics, solubility and a larger therapeutic window.^49^ Furthermore, ADCs with higher DARs can have improved potency because of greater delivery of toxin per antibody bound and may have the potential to eradicate off-target tumor cells, but can suffer from drawbacks including increased plasma clearance and off-target cytotoxicity.^50^

Site-specific engineering may be achieved by integrating additional cysteines or non-natural amino acids with reactive groups for linking payloads.^8^ Thiomab-ADCs, for example, contain engineered cysteines for site-specific conjugation giving a controlled DAR of 2, and have improved homogeneity as well as improved efficacy and toxicity profiles as demonstrated *in vivo* in cynomolgus monkey studies.^51–53^

The trend towards homogenous ADC design enables the emergence of novel bio-orthogonal chemistries that utilize reactive moieties other than thiols or amines, and is broadening the diversity of linking methods.^8^ Shifting the focus by investigating the impact of the antibody scaffold for ADC design might result in future ADCs with: defined DARs, higher solubility, lower off-target toxicity, better-characterized structural and functional attributes and improved efficacy. Therefore, the challenge is to design linking strategies that retain antibody structural integrity and stability, recognition and affinity for the target, Fc-mediated attributes that complement and enhance bioavailability and anti-tumor functions.

## The Fc regions: Antibody scaffold and potential influence on function

The main role of the antibody moiety of an ADC is to deliver the cytotoxic drug selectively to the target cells due to its specificity and high affinity for an antigen expressed on the surface of target cells. Therefore, in designing ADCs, much attention has been paid to the Fab portion of the antibody, responsible for antigen recognition. The antibodies used to develop ADCs are mainly full length recombinant monoclonal antibodies, almost exclusively of the IgG class. Yet the Fc domains of such agents have received less consideration.

### Contributions to efficacy

The Fc portion of IgG antibodies contains the binding domain to the neonatal Fc receptor (FcRn) that regulates serum half-life, and recognition of different activating and inhibitory Fc receptors on immune effector cells that can influence bioavailability, sequestration to tissues, trafficking to tumors, antigen-targeting and immune functions.

An intact IgG ADC might be able to recruit and activate complement components and immune effector cells into the tumor site, mediating secondary immune functions such as complement dependent cytotoxicity (CDC), antibody dependent cell-mediated cytotoxicity (ADCC) and antibody dependent cell-mediated phagocytosis (ADCP).^54^ The ability of ADCs to trigger immune effector functions could offer an advantage through anti-tumor activity, or a disadvantage by sequestering ADCs through immune cells in the circulation and affecting the localization and target cell internalization of ADCs at the tumor site, or by being internalized by immune cells resulting in off-target toxicity. Studies have demonstrated similar antibody-mediated effector functions between the naked antibody and the corresponding ADC. For instance, the capacity of trastuzumab to induce ADCC of breast cancer cells was not affected by conjugation to DM1,^55^ while brentuximab has been described to induce ADCP *in vivo*, believed to contribute to the potent anti-tumor efficacy observed for the brentuximab vedotin ADC.^56,57^ While Fc receptor binding can be advantageous, other studies have identified Fc receptor engagement as a possible cause of side effects of ADC therapeutics. T-DM1 has been demonstrated to be internalized by megakaryocytes *in vivo* via FcγRIIa binding. This has been proposed to be involved in the development of thrombocytopenia induced by T-DM1.^58,59^ However, there are other mechanisms besides FcγRIIa binding, such as macropinocytosis, which could also account for sufficient non-receptor/non-target mediated uptake by megakaryocytes to cause thrombocytopenia.^59^

For these reasons, depending on the type of tumor, the expression of the antigen and the affinity of the antibody for its antigen, it might be appropriate to design an ADC with defined Fc-mediated functions either able or unable to engage with the immune system. ADCs can therefore be designed by selecting an appropriate subclass of IgG or by engineering the Fc portion.

### The impact of antibody subclass

There are 4 subclasses of IgG (IgG1, IgG2, IgG3 and IgG4), and so far all the sub-classes apart from IgG3 have been used to develop ADCs that are currently in clinical trials.^60^

IgG1 is the most commonly used subclass for ADC design. It has comparable serum stability (21 days) to IgG2 and IgG4 but has a greater ability to fix complement and a higher affinity for activating FcγRs expressed on effector cells such as monocytes and macrophages (FcγRI, FcγRIIa, FcγRIIIa) and natural killer (NK) cells (FcγRIIIa).^61^ Therefore, this sub-class has a superior ability to engage the immune system and trigger CDC, ADCC and ADCP.

IgG3 has a superior ability to fix complement and to bind activating FcγRs^61^ but it has so far been avoided for the development of ADCs because of its low half-life in serum compared to the other classes (*e.g.*, 7 days instead of 21 days for IgG1, 2 and 4), its long hinge region that is subject to proteolysis and also evidence of potential immunogenicity.^62^

IgG2 and IgG4 have low or no capacity to fix complement and have lower affinity for the activating FcγRs compared with IgG1. IgG2 and IgG4 are used in the design of therapeutic antibodies when the recruitment of the immune system is not desired. IgG2 has four disulfide bridges, while IgG1 and IgG4 have only two, so it seems more suitable for the use of malemide-linkers allowing a higher DAR.^63^ IgG2 is also able to form dimers, but the impact of dimerization on its therapeutic effects would require more in-depth study. IgG2 ADCs, such as AGS-16M8F (anti-ENPP3 IgG2-MMAF),^64^ are currently under evaluation in clinical trials.^60,65^ IgG4 is a sub-class with very unusual characteristics, such as the ability to undergo Fab arm exchange (FAE) with other IgG4 antibodies, and can thus result in bispecific and functionally monovalent forms with reduced anti-target functions.^66^ Therefore, specific single point mutations (S228P or S228P/R209K)^67,68^ are usually introduced to stabilize the antibody and prevent FAE. Despite lower affinity for the activating FcγRs compared with IgG1, the affinity of IgG4 for FcγRI form is sufficient for functional activation^66^ and in its non-fucosylated form IgG4 is able to bind FcγRIIIa.^69^ Therefore, immune cell activation needs to be considered, when designing ADCs with IgG4 antibodies. The first ADC approved by the FDA in 2000 was an IgG4 antibody (Gemtuzumab ozogamicin, anti-CD33 IgG4-Calicheamicin), withdrawn from the market voluntarily in 2010, since a post approval study showed no improvements in survival and fatal toxicities. However the ADC has been re-approved this year for the treatment of acute myeloid leukemia.^12^ More IgG4 ADCs are currently being evaluated in clinical trials.^60,65^

### Fc domain engineering strategies

Another method to modulate the ability of ADCs to engage the immune system is to engineer their Fc domains. A widely used method to enhance their ability to recruit the immune system and to trigger effector functions is antibody glycoengineering, such as the production of afucosylated IgGs. J6M0-mcMMAF (anti-BCMA IgG1-MMAF) is the first afucosylated-ADC that entered a clinical trial for the treatment of multiple myeloma.^70^

If the sub-class of choice is IgG1, the Fc portion can be engineered to introduce a single point mutation, or a combination of mutations, to enhance or impair IgG1 binding to FcγRs or complement (C1q), and consequently to enhance or impair ADCC, ADCP or CDC.^71–73^ An example is the Phase I clinical trial of MEDI4276 (anti-HER2 IgG1-tubulysin analogue) which has been engineered with three single point mutations (E234F, S239C and S442C) to reduce FcγR binding with the aim to minimize thrombocytopenia seen with T-DM1.^23^

Fc engineering could also be used to improve the pharmacokinetics of ADCs. An example is the humanized IgG1 MEDI-524-YTE engineered with three single point mutations (M252Y, S254T and T256E) to enhance IgG1 binding to the neonatal Fc receptor (FcRn). MEDI-524-YTE has been shown to have a four-fold increase in serum half-life in cynomolgus monkeys compared with the wild type antibody.^74^ It is worth investigating if increased FcRn binding and longer half-life might result in increased activity and decreased toxicity for an ADC.^75^

### ADC design based on biodistribution considerations

Stand-alone small molecule therapeutic agents such as those conjugated to ADCs can be widely distributed in the body. In contrast, antibodies are restricted primarily to plasma and extracellular fluids, and have been shown to target tissues that express the relevant antigen(s). ADCs typically retain the pharmacokinetic properties of their antibody component^3,76^ as opposed to the attached drug, and thus exhibit relatively low clearance and longer half-lives.^77^

Despite the optimization of therapeutic antibodies and the availability of antibodies with higher affinity for the tumor than for normal tissues, the amount of antibody that reaches a tumor is only a small percentage of that administered (e.g., approximately 1–2%).^1,78,79^ For this reason the use of recombinant antibody fragments for ADC production has been evaluated. Antibody fragments such as diabodies are much smaller then IgGs (around 50 kDa versus 150 kDa) and thus have superior tissue penetration abilities. However, due to their smaller size and the lack of the Fc portion that usually binds to FcRn, diabodies are cleared much faster than whole IgG isotypes.^80^ A promising anti-CD30 diabody-drug conjugate has already demonstrated high anti-tumor activity,^81^ but the use of diabodies for ADC design needs further study and optimization with a view to striking a balance between optimum tissue penetration and low clearance rates.

The Fc portion of an ADC is responsible for ADC-mediated effector functions and pharmacokinetics. Therefore, these biological properties can be optimized according to therapeutic requirements via engineering and glycoengineering.

## Conclusion

Over the past decade, research efforts have focused on linker chemistry and optimizing cytotoxic payloads for ADCs. The antibody scaffolds of ADCs provide the required specificity for tumor-associated antigens and can be used to transport prohibitively-cytotoxic agents to cancer tissues. However, the potential clinical effects of the choice of antibody and its structural characteristics remain less-well explored, and perhaps under-exploited. Emerging evidence and novel technologies now provide good reason for a closer consideration of antibody structure, how this is influenced by linking to toxic payloads, and how both the antigen-recognizing Fab regions, as well as the immune-engaging Fc domains, can affect the functions and potency of ADCs (Fig. 1).

In designing the next generation of more effective, specific and efficacious ADCs, we postulate that translational opportunities that can be harnessed by considering the attributes of the antibody component. Careful selection of linking approaches could preserve antibody stability and high target affinities may define and optimize immune cell engagement functions when desired. In the future, ADCs with Fc regions of specific antibody isotypes, and with engineered Fc structure scaffolds may offer better control of biodistribution, and improved immune cell engagement and activation.

Each component of the tripartite complex can be evaluated and optimized to deliver pharmacokinetic and oncolytic properties whilst minimizing off-target toxicities. Novel design approaches informed by antibody structural attributes may thus present opportunities to contribute to the success of the next generation of optimized ADCs.
